# Feed nutritional value of brewers’ spent grain residue resulting from protease aided protein removal

**DOI:** 10.1186/s40104-019-0382-1

**Published:** 2019-09-18

**Authors:** Yizhao Shen, Ranithri Abeynayake, Xin Sun, Tao Ran, Jianguo Li, Lingyun Chen, Wenzhu Yang

**Affiliations:** 1 College of Animal Science and Technology, Hebei Agricultural University, Baoding, 071029 Hebei Republic of China; 2 Agriculture and Agri-Food of Canada, Lethbridge Research and Development Centre, Lethbridge, AB AB T1J4B1 Canada; 3 Department of Agricultural Food & Nutritional Science, University of Alberta, Edmonton, AB T6G 2P5 Canada; 4 College of Food Science and Engineering, Shandong Agricultural University, Taian, 271000 Shandong Republic of China

**Keywords:** Batch culture, Brewers’ spent grain, Chemical composition, Fermentation, Protease hydrolysis

## Abstract

**Background:**

This study was conducted to evaluate the feed nutritional value of brewers’ spent grain (BSG) residue resulting from protease aided protein removal. The nutritional value was measured as nutrient content, gas production, nutrient digestibility and fermentation characteristics in batch culture.

**Results:**

Protein extraction process decreased content of crude protein but concentrated the neutral detergent fibre (NDF) and ferulic acid in BSG residue. The changes in the chemical composition of BSG residue varied with enzyme and enzyme dosage. Digestibility of dry matter (DMD) and NDF of residue differed among proteases. Increasing alcalase dosage linearly decreased DMD, whereas, the DMD linearly increased as everlase or flavourzyme dosage increased. Compared with BSG, the DMD, gas production and fermentation acid concentration of BSG residues were lower, whereas NDF digestibility was higher.

**Conclusions:**

The substantially increased NDF content and improved *in vitro* NDF digestibility due to protease hydrolysis suggest that BSG residue can be potentially exploited as a viable fibre source for ruminant feeding.

## Background

Brewers’ spent grain (BSG) is the major by-product of beer industry, representing ~ 85% of the total by-products generated [1]. Beer is the fifth most consumed beverage in the world, resulting an average annual global production of 39 million tonnes of BSG [1]. Brewing removes the soluble part of the grain, thus concentrating insoluble material in BSG. This includes 15–26% protein and 35–60% fibre on dry basis [2]. Despite the fact that BSG contains significant levels of valuable protein and fibre, its main application is animal feeding, particularly cattle. Nevertheless, there is an increasing research interest in protease hydrolysis of BSG to produce bioactive peptides which could be used for food and nutraceutical applications [3–5]. However, after extraction of peptides, the insoluble residue containing carbohydrate has been treated as waste without any high-value application. Faulds et al. [6] treated BSG with different proteases and found that proteases have the ability to solubilise BSG carbohydrate components in addition to protein. It has been suggested that the carbohydrate bound with protein can be released upon protease hydrolysis [6]. Thus, it is hypothesised that protease hydrolysis of BSG may increase the digestibility of BSG residue, and thus increase its feed value. It is also expected that protease treated BSG may have high fibre content in residue, due to the partial removal of protein.

In addition, it was found that BSG is a good source of phenolic antioxidants including ferulic acid and *p-*coumaric acid [1]. Since most of the phenolic compounds are trapped in the cell walls of cereals, those are not solubilized in brewing, thus get concentrated in BSG [1]. Faulds et al. [6] found that ferulic acid was released upon protease hydrolysis of BSG. Ferulic acid can adversely affect the fibre digestibility [7]. Therefore, the objectives of this study were to prepare BSG residue by protease hydrolysis with different enzymes at different dosages, and evaluate their nutrient digestibility, *in vitro* gas production, and fermentation characteristics using batch culture technique.

## Methods

### Preparation of BSG residue

Three BSG samples from different batches were obtained from a local brewery, and stored at − 20 °C until used. Alcalase (protease from *Bacillus licheniformis*, ≥ 2.4 U/g), everlase (protease from *Bacillus* sp., ≥ 16 U/g), flavourzyme (protease from *Aspergillus oryzae*, ≥ 500 U/g) and viscozyme (carbohydrase from *Aspergillus* sp., 100 Fungal Beta-Glucanase U/g) were purchased from Sigma (St. Louis, USA). Sample was first hydrolyzed with viscozyme to solubilize carbohydrates and then with proteases to release carbohydrates bound to proteins based on the method developed by Xia et al. [8]. In detail, 100 g of BSG was milled to 0.5 mm and solubilized in 500 mL of water to prepare 20% (*w/v*) solution. Then it was kept stirring at 350 r/min for 30 min followed by adjusting to pH 5 and 50 °C which are the optimum conditions for viscozyme. Sample was hydrolyzed with 2% (*w/w*) viscozyme for 1 h, stirring at 350 r/min. Same procedure was followed to prepare nine samples for alcalase, everlase and flavourzyme hydrolysis at three different enzyme to protein ratios (*w/w*): 1:100 (Low), 5:100 (Med), 10:100 (MedHigh), 15:100 (High). Before adding proteases, pH and temperature were adjusted to the optimum of each enzyme, which were pH 8 and 55 °C for alcalase, pH 9.5 and 55 °C for everlase, and pH 6.6 and 55 °C for flavourzyme. The pH and temperature were monitored throughout hydrolysis. After 60 min, samples were heated in a water bath for 20 min at 80 °C to inactivate enzymes. The samples were then kept stirring at 350 r/min for 30 min at pH 10.5 to solubilize proteins. The solubilized proteins were separated from BSG residue by centrifuging at 8000×*g* for 15 min at 20 °C. The precipitate containing insoluble residue was oven dried at 60 °C for 24 h. The oven-dried BSG residues were stored at − 20 °C until *in vitro* evaluation [8].

### Experimental design, substrate and inoculum

The experiment was a complete randomized design with a factorial arrangement of treatments; 3 proteases (alcalase, everlase and flavourzyme) × 4 dosages of each enzyme + BSG (original) with 3 replications (bottles) per treatment combination. The proteases and their dosages were selected based on the preliminary results. The substrates were the BSG residues generated as described above during protein extraction process. The culture was repeated in two runs (batches). Doses of enzyme were expressed as ratio of enzyme to substrate protein (*w/w*), 1:100, 5:100, 10:100, 15:100, respectively, for Low, Med, MedHigh and High. Rumen inoculum was obtained from three ruminally fistulated Angus beef heifers (650 kg of body weight) fed a diet consisting of 60% barley silage, 30% barley straw and 10% protein, vitamin and mineral supplement (DM basis). A high-forage diet was fed to rumen inoculum donor heifers because the substrate was a high-fibre feed. Nutrient composition of the diet was 12.1% crude protein (CP), 50.2% neutral detergent fibre (NDF) and 14.1% starch (DM basis). A total mixed ration was prepared daily using a feed mixer (Data Ranger, American Calan Inc., Northwood, NH, USA) and offered twice daily (morning and afternoon). The animals were cared according to the guidelines of the Canadian Council on Animal Care [9].

### Batch culture procedures

The incubations were performed in triplicate of each treatment combination using glass bottles (100 mL) with rubber stoppers and aluminum caps. Approximately 0.5 g (DM basis) BSG or BSG residue was weighed into acetone-washed and pre-weighed filter bags (F57; porosity: 25 μm, Ankom Technology, Macedon, NY, USA) and hot sealed. Sealed bags were placed in 100 mL fermentation bottle gently. Ruminal contents were collected 2 h before morning feeding from different locations within the rumen and squeezed through four layers of cheesecloth. The squeezed ruminal fluids from 3 animals were mixed and the pH was measured immediately using a pH meter (B20PI, SymHony Benchtop Meters; VWR Edmonton, AB, Canada). The pH of ruminal fluid during this study ranged from 6.5 to 6.8 for the entire experiment. The ruminal fluid was stored in an air tight pre-heated (39 °C) container, transferred to the laboratory within 10 min, and re-strained through 4 layers cheesecloth into a container at 39 °C water bath with bubbling of CO_2_. Forty-five mL McDougall’s buffer and 15 mL ruminal fluid were added into the fermentation bottle, oxygen free CO_2_ was used to flushed and 14-mm butyl rubber stopper and aluminum seal were used to seal the bottle. Sealed bottles were placed on an oscillating shaker (125 r/min of oscillation speed) in a 39 °C incubator for 24 h incubation. Triplicates were used for every sample in each run. Two runs were carried out within 2 weeks. Additionally, three blank controls with empty Ankom bags only were used to correct gas production in each run.

Gas pressure was recorded at 3, 6, 9, 12 and 24 h of incubation using a pressure transducer (model PX4200-015GI, Omega Engineering, Inc., Laval, QC, Canada) with a 23 gauge needle (0.6 mm) through rubber stoppers. Then gas was vented by leaving the needle in place and removing the transducer. Pressure values, corrected for the gas released from the blanks, were used to generate volume estimates using the equation of Mauricio et al. [10]: GP_t_ (mL) = 0.18 + 3.697P_t_ + 0.0824P_t_^2^, where GP_t_ is the gas production volume at time ‘t’ (h), P_t_ is the gas pressure measured at time ‘t’ (h).

Kinetics of gas production was generated by fitting gas production data to an exponential model [11] as: y = GV × (1 – e^– c × (t – lag)^), where ‘y’ is the cumulative volume of gas produced at time ‘t’ (h), ‘GV’ is the asymptotic gas volume, ‘c’ is the gas production rate and ‘lag’ is the time (h) between inoculation and commencement of gas production.

After 24 h of incubation, fermentation bottles were placed into ice to stop the fermentation. Fermentation media pH was measured immediately using a pH meter (B20PI, SymHony Benchtop Meters; VWR Edmonton, AB, Canada). Subsample of 5 mL fermentation liquid was mixed with 1 mL 25% (*w/v*) HPO_3_ for volatile fatty acid (VFA) analysis and another 5 mL fermentation media was mixed with 1 mL 1% (*w/v*) H_2_SO_4_ solution used for NH_3_-N analysis. The bags were removed from the bottles, washed manually under tap water and oven dried at 55 °C for 48 h to determine the DM digestibility (DMD). The DMD was calculated by the weight loss of the substrate.

### Chemical analyses

Samples of BSG and BSG residue before and after incubation were analyzed for DM (903.15), acid detergent fibre (ADF; 973.18) and ash (942.05) according to the standard methods of AOAC [12]. The OM content was calculated as 100 minus ash content. The total N content in the BSG and BSG residue was analyzed using flash combustion and thermal conductivity detection technique (model 1500, Carlo Erba Instruments, Milan, Italy) and CP was calculated as N × 6.25. The NDF content in the BSG and BSG residue before and after incubation was determined according to Larbi et al. [13] using heat-stable α-amylase (Termamyl 120 L, Novo NordiskBiochem, Franklinton, NC, USA) with sodium sulfite. The NDF and ADF contents were expressed with ash inclusion. Hemicellulose was calculated as the difference between NDF and ADF. Non-fibre carbohydrate (NFC) was calculated as OM content minus the sum of NDF, ether extract and CP contents. The concentration of VFA in fermentation media was analysed using a gas chromatograph (model 5890, Hewlett-Packard Lab, Palo Alto, CA, USA) equipped with a capillary column (30 m × 0.32 mm i.d., 1-μm phase thickness, Zebron ZB-FAAP, Phenomenex, Torrance, CA, USA) and flame ionization detection. The concentration of NH_3_-N in fermentation media was determined as described by Rhine et al. [14]. Total ferulic acid content in BSG and BSG residue was analysed as described by Cao et al. [15] using HPLC with a Symmetry reverse phase C-18 column (250 mm × 4.6 mm i.d., 5-μm phase thickness; Waters, Milford, MA, USA).

### Statistical analyses

Data were analysed using the MIXED procedure of SAS (SAS Inst. Inc., Cary, NC, USA) with model including enzymes, enzyme dosages and the interaction as fixed effects, replicates of BSG and BSG residue extracted by same enzyme and different runs were considered as random effects. The effect of increasing enzyme dosages was examined through linear and quadratic orthogonal contrasts using the CONTRAST statement of SAS. Contrasts were generated to compare the average of four enzyme dosages and original BSG. The PDIFF option adjusted by the Turkey method was included in the LSMEANS statement to account for multiple comparisons among treatments. Differences between treatments were declared significant at *P* ≤ 0.05. Trends were discussed at 0.05 < *P* ≤ 0.10. The Pearson correlation coefficient was analysed with the CORR procedure of SAS (SAS Inst. Inc., Cary, NC, USA).

## Results & discussion

### Effect of protease hydrolysis on chemical composition of BSG residue

The BSG is considered as a lignocellulosic material rich in protein and fibre, which may vary substantially, ranging from 15% to 35% and 43% to 73%, respectively [16–18]. The considerable variation in the chemical composition of BSG among studies could be due to differences in barley variety, harvest time, malting and mashing conditions, and the quality and type of additives added in the brewing process [19]. The CP (25.6%) and NDF (46.6%) contents of the BSG used in our protein extraction is within the range reported in the literature.

There were interactions between enzyme and enzyme dosages on the contents of OM, NDF, ADF and CP of BSG residues; although statistically significant, the differences in the OM, NDF and ADF contents of BSG residue among enzymes were quantitatively small (Table 1). However, the CP content of the residue treated with flavourzyme was greater (*P <* 0.01) than the alcalase or everlase-treated residues regardless of the enzyme dosages used. In addition, the CP content of the residue treated with everlase was also greater (*P <* 0.05) than acalase-treated residue at Low or Med dose. Celus et al. [20] used different enzymes to generate BSG protein hydrolysates and found that both alcalase and flavourzyme improved the protein solubility, whereas the alcalase seemed to have greater activity than flavourzyme [20]. Therefore, this is a likely explanation for greater protein content left over in the BSG residue treated with flavourzyme than with alcalase. Though the use of everlase to hydrolyse BSG protein was seldom reported, the changes of CP content in BSG residue in present study illustrated that alcalase may have greater activity in protein solubility than everlase at Low or Med dose but both enzymes had the similar activity at MedHigh and High dose. The small quantitative differences in the content of OM and NDF with increasing enzyme dose in present results are consistent with previous studies that showed often little effect of protease dose-response on BSG solubility [21]. Treimo et al. [22] reported that a reduction of the peptidase dosages from 20 to 5 μL/g DM reduced DM and protein yields of BSG residue only from 38% to 36% and from 72% to 67%, respectively. The marginal gain on BSG DM and protein solubility obtained by considerable increases in alcalase dosages from 1.2 to 20 μL was also limited in the study of Treimo et al. [23]. These results suggest that the protease dosage attributed limited effects on solubilizing DM and protein of BSG.

**Table 1:** Chemical composition (% of DM) of brewers’ spent grain (BSG) and residues after hydrolysis using proteases varying with enzyme doses

Item	Residue, enzyme dose^d^				BSG	SEM	*P <*				
	Low	Med	MedH	High			Enz	L	Q	Int	R:BSG
OM											
Alcalase	95.8^a^	95.2^a^	94.3	94.2	94.4	0.23	0.01	0.01	0.14	0.02	0.03
Everlase	94.2^b^	94.1^b^	93.9	94.0	94.4			0.45	0.72		0.18
Flavourzyme	94.5^b^	94.3^b^	94.0	94.2	94.4			0.16	0.41		0.67
NDF											
Alcalase	71.4	72.7	70.9	74.8^a^	46.6	0.74	0.01	0.01	0.08	0.05	0.01
Everlase	71.6	72.6	69.2	75.0^a^	46.6			0.05	0.01		0.01
Flavourzyme	69.7	69.0	70.1	71.4^b^	46.6			0.06	0.21		0.01
ADF											
Alcalase	30.8^a^	31.4^a^	31.1^a^	32.1	20.8	0.43	0.01	0.07	0.70	0.02	0.01
Everlase	28.5^b^	29.0^b^	29.1^b^	31.5	20.8			0.01	0.05		0.01
Flavourzyme	28.9^b^	28.2^b^	30.0^a^	31.0	20.8			0.01	0.11		0.01
HC											
Alcalase	40.6	41.2^b^	39.8	42.6^a^	25.8	0.71	0.01	0.12	0.10	0.12	0.01
Everlase	43.1	43.6^a^	40.0	43.5^a^	25.8			0.44	0.02		0.01
Flavourzyme	40.8	40.8^c^	40.1	40.4^b^	25.8			0.52	0.75		0.01
NFC											
Alcalase	9.1^a^	8.9^a^	9.2^a^	6.2^a^	18.1	0.82	0.01	0.03	0.09	0.01	0.01
Everlase	5.9^b^	6.4^b^	10.1^a^	6.0^a^	18.1			0.30	0.01		0.01
Flavourzyme	0.5^c^	0.7^c^	0.2^b^	1.5^b^	18.1			0.43	0.47		0.01
FA											
Alcalase	0.35	0.39^ab^	0.39^a^	0.43	0.30	0.018	0.01	0.02	0.92	0.12	0.01
Everlase	0.34	0.43^a^	0.35^b^	0.40	0.30			0.19	0.32		0.01
Flavourzyme	0.32	0.34^b^	0.30^c^	0.35	0.30			0.32	0.32		0.21
CP											
Alcalase	9.3^c^	7.3^c^	8.0^b^	6.8^b^	25.6	0.49	0.01	0.01	0.41	0.01	0.01
Everlase	10.9^b^	9.2^b^	8.9^b^	6.7^b^	25.6			0.01	0.76		0.01
Flavourzyme	18.6^a^	19.0^a^	17.7^a^	15.0^a^	25.6			0.01	0.01		0.01

*OM* organic matter, *NDF* neutral detergent fibre, *ADF* acid detergent fibre, *HC* hemicellulose, *NFC* non-fibre carbohydrate, *FA* ferulic acid, *CP* crude protein

Enz, enzyme effects; L, Q, linear and quadratic effects of enzyme dose (enzyme:protein, *w/w*; 1:100, 5:100, 10:100, 15:100, respectively, for Low, Med, MedH and High); Int, interaction between enzyme and enzyme dosage; R:BSG, contrast between average of enzyme dosage and BSG

^a, b, c^Means with different letters in the same column and within a category differed (*P <* 0.05)

^d^Protease dosage used in protein hydrolysis (% of BSG protein)

Ferulic acid content in BSG residue differed among enzymes at Med and MedHigh enzyme dose, and linearly (*P <* 0.02) increased with increasing alcalase dose, whereas it was not affected with increasing the dosages of everlase or flavourzyme (Table 1). Total ferulic acid content increased (*P <* 0.01) in BSG residue treated with alcalase or everlase compared to the BSG, but no difference was observed between BSG and BSG residue treated with flavourzyme. Ferulic acid is the phenolic acid mainly existed in plant cell walls, usually links with arabinoxylans, and provides more enzyme resistance in fibre digestion [7]. Ikram et al. [2] reported 0.34% of ferulic acid in BSG, which is similar to the value of 0.30% observed in the present study. Since ferulic acid is mainly associated with hemicellulose, the greater content of hemicellulose in BSG residue resulted with greater ferulic acid content in present study was expected. However, the hemicellulose content increased by 60% in BSG residue vs. BSG (average 41.4% vs. 25.8%), while the ferulic acid content increased only by 22% in the BSG residue vs. BSG (average 0.36% vs. 0.30%). Previous studies recovered that alcalase had the activity to release ferulic acid from BSG [24, 25]. Flavourzyme was also reported to have high activity in hydrolysis of the ferulate [26]. It suggests that a portion of ferulic acid in BSG was loss as soluble ferulic acid due to protease hydrolysis.

During protease hydrolysis of BSG, the NDF content was concentrated, while the CP and NFC content considerably reduced in the BSG residue compared with BSG without obviously changing the OM content (Table 1). The NFC in BSG could be mainly the starch and some oligosaccharide like glucose or maltose, which should be readily digestible. The dramatically decreased content of NFC in the residue compared with BSG suggested that the proteases might have carbohydrase activity, particularly for flavourzyme, in which the NFC in the residue was mostly gone. Faulds et al. [6] found considerable reduction of oligomers in protease-treated BSG. Xiros and Christakopoulos [27] also reported that alcalase combined with other enzymes, hydrolysed up to 36% BSG into soluble fraction and monosaccharides.

### Effect of protease hydrolysis on digestibility of BSG and BSG residue

**Fig. 1:**
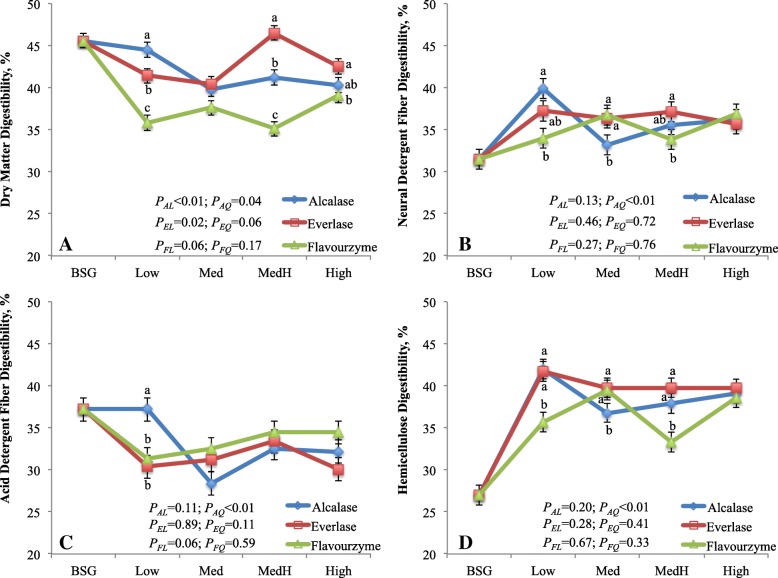
*In vitro* digestibility of brewers’ spent grain and residues treated by proteases varying with dosages. ^a-c^ Means with different letters with the same dose of enzyme are different (*P* < 0.05). BSG, brewers’ spent grain; AL, EL, FL are linear and AQ, EQ, FQ are quadratic effects, respectively, for alcalase, everlase and flavouezyme dose (enzyme:protein, *w/w*; 1:100, 5:100, 10:100 and 15:100, respectively for Low, Med, MedH and High). All enzyme × enzyme dosage and the contrasts between the average of 4 doses of each enzyme and BSG were significant (*P* < 0.01). The enzyme effects were *P* < 0.01, *P* < 0.38, *P* < 0.18 and *P* < 0.01 for Figure **a**, **b**, **c** and **d**, respectively

There was consistent interaction between enzyme and enzyme dosage on the digestibility of DM, NDF and ADF (Fig. 1). The DMD was greatest in the residue treated with alcalase, intermediate with everlase and lowest with flavourzyme at Low dose (*P <* 0.01), whereas it was greatest with everlase, intermediate with alcalase and lowest with flavourzyme at MedHigh dose (*P <* 0.01). With increasing alcalase doses, the DMD linearly (*P <* 0.01) decreased, whereas it linearly (*P <* 0.02) increased as everlase dose increased. The DMD of BSG residue was lower (*P <* 0.01) than that of BSG (40.4% vs. 45.6%). Digestibility of NDF was greater (*P <* 0.02) in the residue treated with alcalase than flavourzyme at Low dose; it was lower (*P <* 0.02) with alcalase than everlase or flavourzyme at Med dose; and it was greater (*P <* 0.05) with everlase than flavourzyme at MedHigh dose with no difference at High dose. The ADF digestibility was different among enzymes only at Low dose. With increasing alcalase doses, the digestibility of NDF and ADF quadratically (*P <* 0.01) decreased, whereas no response of everlase or flavourzyme dose was observed on the digestibility of NDF and ADF. The digestibility of NDF was greater (*P <* 0.01) but that of ADF was lower (*P <* 0.01) for BSG residue than for BSG. The DMD is the average digestibility of individual nutrient including primarily NFC (starch, sugars), fibre and CP. In the present study, the lower DMD of BSG residue than that of BSG was mainly due to decreased NFC (residue vs. BSG; average 5.5% vs. 18.1%) and CP content (residue vs. BSG; average 11.5% vs. 25.6%) in the residue. Although the NDF digestibility of residue was greater, it was offset by the lower ADF digestibility compared with that of BSG. Similarly, the overall lowest DMD of flavourzyme treated residue was consistent with the lowest NFC content of the residue. The NFC is readily digestible, CP is the next and fibre is lowest digestible nutrient in the digestive tract of ruminants [28]. This was further supported by Pearson correlation analysis that the DMD was positively correlated with NFC content (*r* = 0.75; *P <* 0.01; Table 2), but it moderately and negatively related to the NDF content (*r* = − 0.49, *P <* 0.01).

**Table 2:** Pearson correlation coefficient between chemical composition and feed digestibility

Item	1	2	3	4	5	6	7
1. DMD, %							
2. NDFD, %	0.16						
3. HCD, %	−0.11	0.87^**^					
4. NDF, % DM	−0.49^**^	0.50^**^	0.82^**^				
5. HC, % DM	−0.50^**^	0.51^**^	0.83^**^	0.99^**^			
6. Total FA, % DM	−0.06	0.37^*^	0.62^**^	0.70^**^	0.68^**^		
7. NFC, % DM	0.75^**^	−0.36^*^	−0.60^**^	−0.81^**^	− 0.82^**^	− 0.25^*^	
8. CP, % DM	0.12	− 0.47^**^	− 0.76^**^	− 0.87^**^	− 0.84^**^	−0.88^**^	0.41^**^

*DMD* dry matter digestibility, *NDF* neutral detergent fibre, *NDFD* neutral detergent fibre digestibility, *FA* ferulic acid, *HC* hemicellulose, *HCD* hemicellulose digestibility, *NFC* non-fibre carbohydrate, *CP* crude protein

^*^P < 0.05, ^**^P < 0.01

The method that developed for NDF analysis in animal nutrition is to determine the fibre for its biological nature rather than for chemical nature, thus the NDF is mainly composed of hemicellulose, cellulose and lignin. The hemicellulose has greater digestibility than cellulose, and lignin is normally indigestible in the digestive tract of livestock animals [29]. Therefore, the improved NDF digestibility of the residue versus BSG was particularly due to greater hemicellulose digestibility in residue (39%) than in BSG (27%) as well as slightly greater hemicellulose proportion in the residue than in BSG (58 vs. 55% of NDF). The NDF digestibility is highly correlated with the digestibility of hemicellulose (*r* = 0.87, *P <* 0.01; Table 2). The improved hemicellulose digestibility was likely resulted from partly release of ferulic acid from residue during protease extraction. In BSG, arabinoxylans is one of the main components of hemicellulose and contain large amount of ferulic acid [30]. Ferulic acid can esterify to arabinose, enlarge the molecular weight of arabinoxylans, and adversely affect the digestibility and solubility of arabinoxylans [7, 31]. However, the reduction in ADF digestibility of the residue compared with BSG is not clear in present study. We speculate that the residue containing low NFC might decrease bacterial colonization on the residue, which is a critical step for the fibre digestion in the rumen [32].

**Fig. 2:**
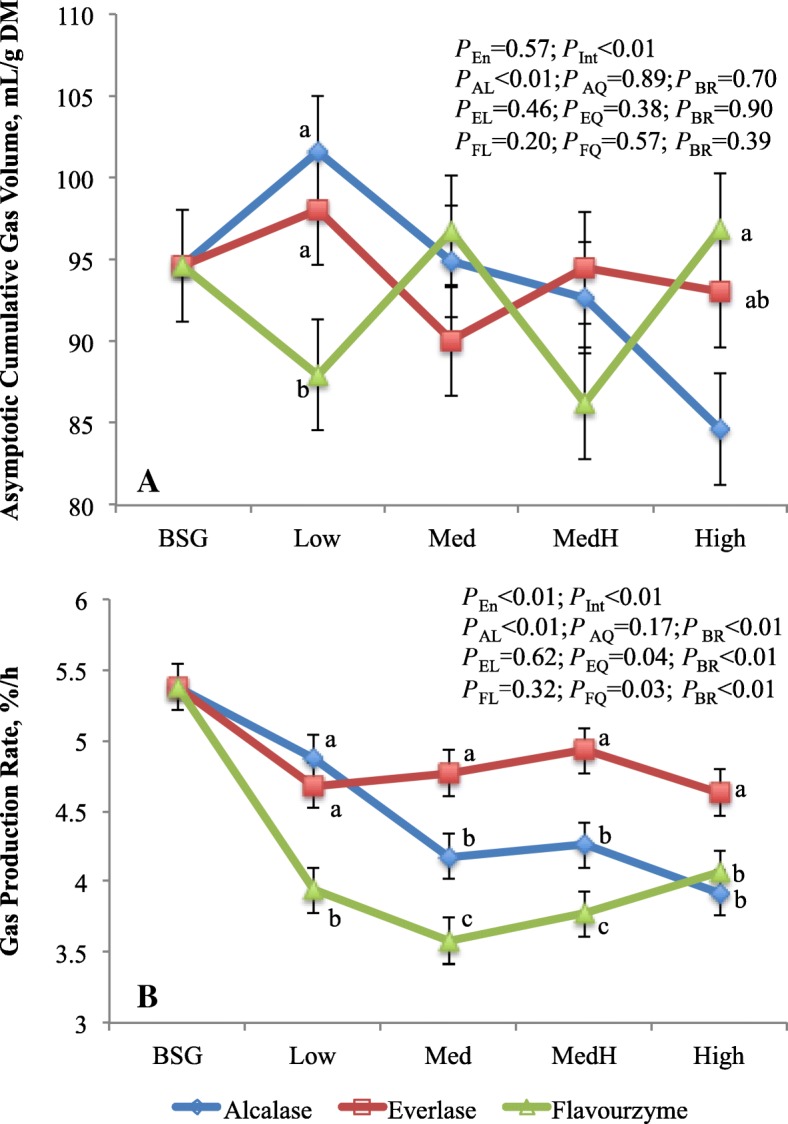
Asymptotic gas volume (mL/g DM; **a**) and gas production rate (%/h; **b**) of brewers’ spent grain and residues treated by proteases varying with dosages. ^a-c^Means with different letters in the same dose are different (*P* < 0.05). BSG, brewers’ spent grain; *P*_en_, enzyme effects; *P*_Int_, interaction between enzyme and enzyme dosage; *P*_AL_, *P*_EL_, *P*_FL_ are linear and *P*_AQ_, *P*_EQ_, *P*_FQ_ are quadratic effects of, respectively, alcalase, everlase and flavouezyme dose (enzyme:protein, *w/w*; 1:100, 5:100, 10:100 and 15:100, respectively for Low, Med, MedH and High); *P*_BR_, contrast between average of 4 doses of each enzyme and BSG

Interaction of enzyme with enzyme dose on gas production kinetics was noticed, which is consistent with the digestibility results (Fig. 2). The asymptotic gas volume was lower (*P <* 0.04) at Low dose but it was greater (*P <* 0.05) at High dose with flavourzyme-treated residues compared to alcalase or everlase-treated residues. Overall, gas production rate was greatest (*P <* 0.01) in residue treated with everlase, intermediate with alcalase and lowest with flavourzyme. Increasing alcalsae dose linearly (*P <* 0.01) decreased asymptotic gas volume and gas production rate, while the gas production rate was quadratically (*P <* 0.04) changed as everlase or flavourzyme doses increased. The asymptotic gas volume did not differ between residue (93.1) and BSG (94.6 mL/g DM), whereas the gas production rate was lower (*P <* 0.01) for residue (4.30%/h) than BSG (5.38%/h). In gas production system, the asymptotic gas volume and gas production rate reflect the extent and rate of the substrate fermentation, respectively, and the gas production *in vitro* is highly correlated to substrate digestion [33]. The linearly decreased gas production was consistent with the linear reduction of DMD with increasing alcalase doses. However, contrary to the DMD, the asymptotic gas volume of everlase or flavourzyme-treated residues did not show the enzyme dose response. This may be explained by differences in other fermentation products because the truly digested substrates in batch culture are divided among VFA, gas, and microbial biomass. The gas production rate is highly correlated with the NFC content of the substrates [34], therefore, the similar responses to enzyme and enzyme dose between NFC content and gas production rate were observed.

### Effect of protease hydrolysis on fermentation patterns of BSG and BSG residue

The enzyme and enzyme dose interaction was noticed on VFA concentration, molar proportion of acetate, propionate and ratio of acetate to propionate (A:P; Table 3). The VFA concentration was greater (*P <* 0.05) in residue treated with alcalase than with flavourzyme at Low dose, which agreed with the DMD response. The VFA concentration had the similar enzyme dose response as to that observed for the DMD. In addition, the lower (*P <* 0.01) VFA concentration for the residue than BSG is also in accordance with lower DMD of the residue. The differences in molar proportion of individual VFA were quantitatively small among enzymes even though they were statistically significant. The greater proportion of acetate with residue than with BSG might be explained by the greater NDF digestibility of the residue. The A:P in residue treated with flavourzyme was greater (*P <* 0.05) than with alcalase at Low dose and than with everlase at MedHigh dose, and it quadratically (*P <* 0.01) increased with increasing alcalase or flavourzyme doses. The A:P was also greater (*P <* 0.01) with flavourzyme-treated residue than BSG. Overall, the A:P of BSG residue appeared to be low considering its high NDF content (> 70% of DM). The low A:P could be due to high hemicellulose content along with its high digestibility in the residue. Murphy et al. [35] reported that the fermentation of hemicellulose by ruminal microbes resulted in a greater reduced A:P than with the fermentation of cellulose. The low A:P indicates relatively greater propionate proportion, and thus greater fermentation efficiency [36]. When propionate is produced, the carbon and hydrogen from glucose are still present in VFA, whereas when acetate is produced, the part of carbon and hydrogen from glucose are produced as CO_2_ and methane. Therefore, lack of difference in A:P between BSG and residue treated with alcalase and everlase suggested the fermentation efficiency of the residue was not reduced.

**Table 3:** *In vitro* fermentation characteristics of brewers’ spent grain (BSG) and residues after hydrolysis using proteases varying with enzyme dosages

Item^e^	Residue, enzyme dose^d^				BSG	SEM	*P <*				
	Low	Med	MedH	High			Enz	L	Q	Int	R:BSG
VFA, mmol/L											
Alcalase	64.1^a^	60.1	55.0^b^	57.1	67.0	2.49	0.55	0.01	0.03	0.01	0.01
Everlase	58.1^ab^	59.5	62.3^a^	61.6	67.0			0.06	0.40		0.01
Flavourzyme	55.8^b^	61.6	60.0^ab^	62.6	67.0			0.01	0.30		0.01
Acetate (A), %											
Alcalase	56.5^b^	57.1	57.6^ab^	57.2	56.4	1.07	0.01	0.01	0.01	0.01	0.01
Everlase	57.7^a^	57.6	57.1^b^	57.4	56.4			0.09	0.21		0.01
Flavourzyme	57.8^a^	57.9	58.5^a^	58.0	56.4			0.10	0.04		0.01
Propionate (P), %											
Alcalase	23.1^a^	21.6^b^	21.6^b^	21.7	21.9	0.49	0.01	0.01	0.01	0.01	0.36
Everlase	22.1^b^	22.1^a^	22.2^a^	22.0	21.9			0.94	0.59		0.20
Flavourzyme	21.6^c^	21.5^b^	21.1^c^	21.8	21.9			0.43	0.01		0.01
Butyrate, %											
Alcalase	2.18	2.21	2.19	2.20	2.14	0.043	0.10	0.59	0.73	0.87	0.05
Everlase	2.20	2.19	2.15	2.18	2.14			0.36	0.47		0.13
Flavourzyme	2.15	2.16	2.16	2.14	2.14			0.87	0.59		0.60
A:P											
Alcalase	2.46^b^	2.66	2.67^ab^	2.65	2.59	0.128	0.01	0.01	0.01	0.01	0.31
Everlase	2.63^ab^	2.64	2.59^b^	2.62	2.59			0.34	0.48		0.16
Flavourzyme	2.70^a^	2.71	2.79^a^	2.68	2.59			0.95	0.01		0.01
NH_3_-N, mmol/L											
Alcalase	24.1	24.1	23.3	24.1	22.6	1.75	0.51	0.75	0.51	0.85	0.09
Everlase	24.1	23.9	24.4	24.1	22.6			0.85	0.93		0.05
Flavourzyme	23.9	24.2	23.6	22.4	22.6			0.10	0.35		0.23

*VFA* volatile fatty acid, *BCFA* branched chain fatty acid

Enz, enzyme effects, L, Q, linear and quadratic effects of enzyme dose (enzyme:protein, *w/w*; 1:100, 5:100, 10:100, 15:100, respectively, for Low, Med, MedH and High); Int, interaction between enzyme and enzyme dosage; R:BSG, contrast between average of enzyme dosage and BSG

^a, b, c^Means with different letters in the same column and within a category are different (*P <* 0.05)

^d^Protease dosage used in protein hydrolysis (% of BSG protein)

The concentration of NH_3_-N in fermentation media was not affected by the treatments except that the concentration of NH_3_-N was slightly greater with alcalase (23.9 mmol/L; *P <* 0.09) and everlase (24.1 mmol/L; *P <* 0.05) treated residue than BSG (22.6 mmol/L; Table 3). The NH_3_-N concentration from this study was similar to the previous report in batch culture incubated high-grain substrate (90% barley DM) [34]. Rumen NH_3_-N concentration is dynamic balance between production from proteolysis and utilization for microbial protein production. Carbohydrate availability strongly influences the utilization of NH_3_-N by rumen microbes. Benchaar et al. [37] observed lower ruminal NH_3_-N concentration when the starch supply increased in the diet. Therefore, the lower NH_3_-N concentration with BSG may be explained by the greater NFC content of BSG, which might increase the use of NH_3_-N by microbes. However, the lack of the NH_3_-N concentration response to enzyme or enzyme dose was somehow not expected. For example, the higher NH_3_-N concentration could be higher for flavourzyme-treated residue because of its higher CP contents and lower NFC content. It speculates that protein degradability of flavourzyme residue may be low.

## Conclusions

In summary, the protease hydrolysis increased the fibre and ferulic acid content of the BSG residue, but decreased NFC and CP content. Although the DMD and total VFA concentration decreased, higher NDF digestibility was observed for the residue compared with BSG. The alcalase and everlase overall demonstrated better hydrolysis efficiency on protein removal and generated greater DMD in the residue when compared to flavourzyme. Increasing dosages of alcalase and everlase also increased fermentability of the residue. These results indicated that the overall feed value of the BSG residue may be slightly lower than BSG, it is a viable fibre source in ruminant feeds. In addition, the higher ferulic acid content of the residue is warranted further study for its antioxidant activity.

## Data Availability

The data analyzed during the current study are available from the corresponding author on reasonable request.

## Abbreviations

A: P ratio of acetate to propionate
ADF: Acid detergent fibre
BSG: Brewers’ spent grain
CP: Crude protein
DM: Dry matter
DMD: DM disappearance
Med: Medium
MedHigh: Medium high
NDF: Neutral detergent fibre
NFC: Non-fibre carbohydrate
VFA: Volatile fatty acid
